# The complete plastome of *Garcinia subelliptica,* Merr. 1909 (Clusiaceae)

**DOI:** 10.1080/23802359.2022.2029603

**Published:** 2022-02-03

**Authors:** Pei-Dong Chen, Da-Juan Chen, Xiu-Rong Ke, Zhi-Xin Zhu, Hua-Feng Wang

**Affiliations:** Hainan Key Laboratory for Sustainable Utilization of Tropical Bioresources, College of Tropical Crops, Hainan University, Haikou, China

**Keywords:** *Garcinia subelliptica*, Merr. 1909, Clusiaceae, complete plastome, phylogenetic relationships

## Abstract

The complete plastome of *G. subelliptica*, Merr. 1909. The complete length is 158,356 bp, with the typical structure and gene content of angiosperm plastomes, including a large single-copy region (LSC) of 86,220 bp, a repeat region (IRB), and a reverse repeat region (IRA) of 27,399 bp, respectively, and a small single-copy region (SSC) of 17,338 bp. The plastome contains 130 genes, including 85 protein-coding genes, 37 tRNA genes, and 8 rRNA genes. The total G/C content of the plastome is 36.1%.

## Introduction

*Garcinia subelliptica*, Merr. 1909 is a tree species belonging to the tribe Garcinieae within the family Clusiaceae. The species is distributed in southern Taiwan of China, Japan’s Ryukyu Islands, the Philippines, Sri Lanka, and Indonesia (Java). Suitable habitats for the species can be found in mixed-wood forests along the seashore. The type specimen is from the Philippines. The species serves as an ideal tree species for constructing windbreaks in coastal areas of China. Based on systematic studies of the chemical composition and structural identification of the fruits, 59 compounds were isolated and identified, with nine showing potential antitumor activity (Yongling 2019). Several active compounds are found in the fruit, seeds, leaves, wood, bark, and roots of individual trees. The main compounds are benzone, xanthone, diflavone, and triterpenoid. Garsubellin A was isolated from the wood and has the potential for the development of treatments for Alzheimer’s disease by displaying anti-inflammatory properties to inhibit the disease. Therefore, we report a complete plastome of *G. subelliptica*, Merr. 1909 to improve the quality of related collections, medical applications, and phylogenetic studies of Clusiaceae.

In this study, the leaves of *G. subelliptica,* Merr. 1909 (MZ929421) were sampled from Hainan Ledong (116.40°E, 39.96°N). A voucher specimen (voucher code: X.-F. Zhang, A277, HUTB) and its DNA were deposited in the Herbarium of the Institute of Tropical Agriculture and Forestry (code of herbarium: HUTB), Hainan University, Haikou, China. A specimen was deposited at Hainan University (https://ha.hainanu.edu.cn/home2020/, H.-F. Wang and hfwang@hainanu.edu.cn) under the voucher number Wang et al. A277.

Total genomic DNA was extracted from dried leaf tissue using the cetyltrimethyl ammonium bromide (CTAB) protocol of Doyle and Doyle (1987). Cleaned sequence data was assembled with GetOrganelle v1.7.5.0 (Jin et al. 2020). The plastome was annotated against the plastome of *Garcinia pedunculata* (NC048983) by using Geneious v2021.1.1 (Biomatters Ltd., Auckland, New Zealand) and the annotation was corrected with DOGMA (Wyman et al. 2004).

Our results show that the complete length of the plastome is 158,356 bp, with the typical structure and gene content of angiosperm plastomes, including a large single-copy region (LSC) of 86,220 bp, a repeat region (IRB) of 27,399 bp, and a reverse repeat region (IRA) of 27,399 bp, and a small single-copy region (SSC) of 17,338 bp. The plastome contains 130 genes, including 85 protein-coding genes, 37 tRNA genes, and 8 rRNA genes. The overall G/C content of the plastome in *G. subelliptica*, Merr. 1909 is 36.1%, with the G/C content being 33.5% in the LSC, 30.2% in the SSC, 42.0% in IRA, and 42.0% in IRB, respectively.

**Figure 1.**
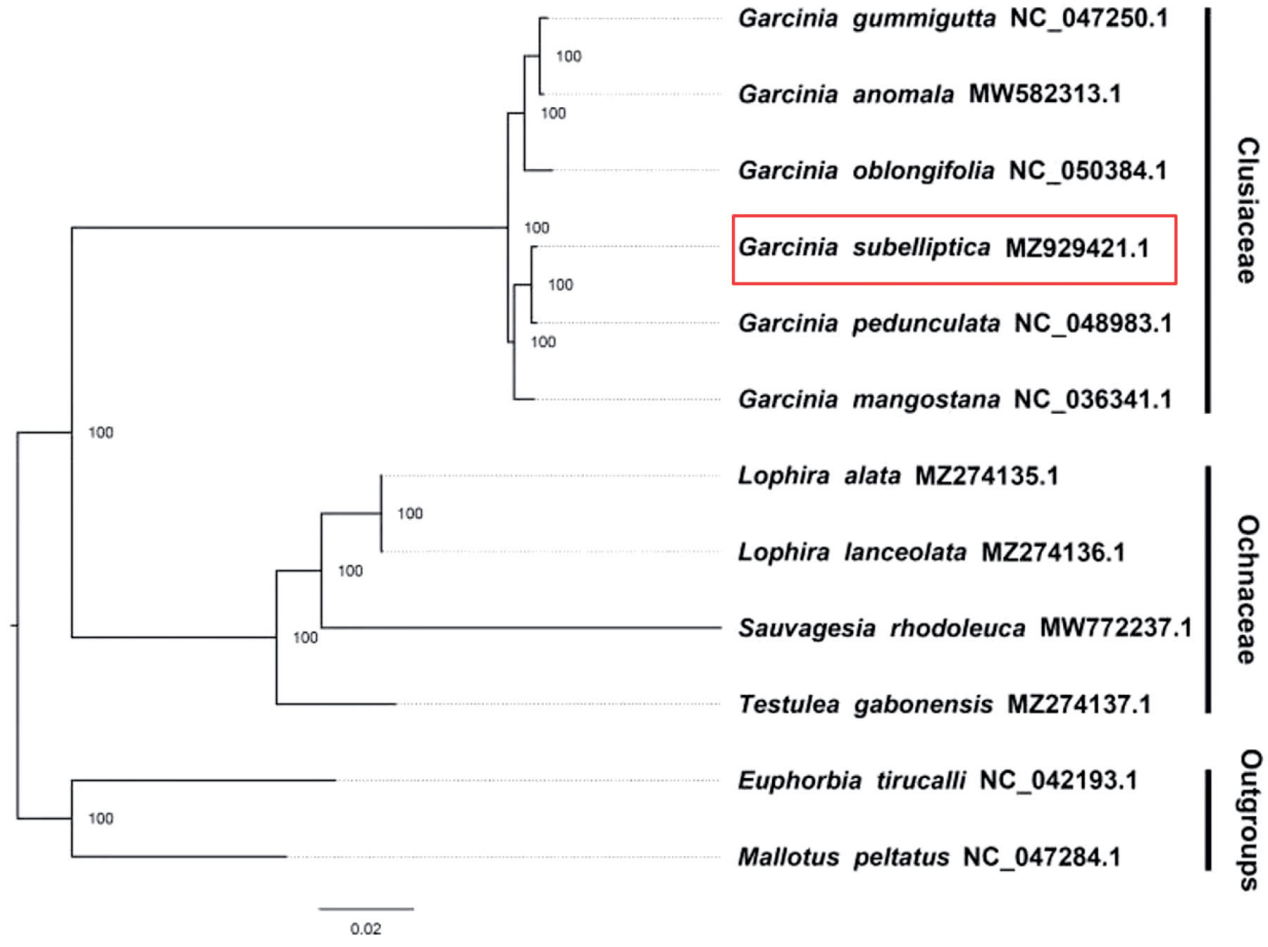
The maximum-likelihood phylogeny obtained from 12 complete plastome sequences using RAxML.

We included sequences from NCBI whose phylogenetic relationships are closely related. *Euphorbia tirucalli*, L 1753 (NC042193.1) and *Mallotus peltatus*, (Geiseler) Müll.Arg. 1865. (NC047284.1) were selected as outgroups. Sequences were aligned with MAFFT. Phylogenetic relationships were inferred with Maximum Likelihood using the aligned sequences using the CIPRES portal (https://www.phylo.org/portal2/login!input.action). We found that *G. subelliptica*, Merr. 1909 is more closely related to *G. pedunculata* than other taxa in the phylogenetic tree (Figure 1). These results promote the protection and phylogenetic research of *G. subelliptica*, Merr. 1909, promoting the value of utilization and development of *G. subelliptica*, Merr. 1909 in Chinese medicine, deepening the understanding of Clusiaceae.

## Data Availability

Data supporting the results of the study of genome sequence are in the NCBI GenBank (https://www.ncbi.nlm.nih.gov/) with registration number MZ929421, which are publicly available. The related BioProject, raw sequencing files in SRA, and the Bio-Sample number are PRJNA748537, SRR15498084 and SAMN20607948, respectively.
